# Effects of Aspirin in Rats With Ouabain Intracerebral Treatment—Possible Involvement of Inflammatory Modulation?

**DOI:** 10.3389/fpsyt.2019.00497

**Published:** 2019-07-16

**Authors:** Lin Zhang, Li-Ting An, Yan Qiu, Xiao-Xiao Shan, Wen-Li Zhao, Jing-Ping Zhao, Le-Hua Li, Bing Lang, Ren-Rong Wu

**Affiliations:** ^1^Department of Psychiatry, The Second Xiangya Hospital, Central South University, Changsha, China; ^2^National Clinical Research Center for Mental Disorders, Changsha, China; ^3^School of Medicine, Medical Sciences & Nutrition, Institute of Medical Science, University of Aberdeen, Aberdeen, United Kingdom; ^4^Hunan Key Laboratory of Animal Models for Human Diseases, Central South University, Changsha, China; ^5^Shanghai Institutes for Biological Sciences, Chinese Academy of Sciences, Shanghai, China

**Keywords:** mania, ouabain, cytokine, toll-like receptor, aspirin, animal model

## Abstract

Bipolar disorder (BD) is a chronic and refractory disease with high probability of morbidity and mortality. Although epidemiological studies have established a strong association between BD and immune dysfunction, the precise etiology is still debatable, and the underpinning mechanism remains poorly investigated and understood. In the present study, manic-like symptoms of BD were induced in rats after intracerebroventricular administration of ouabain. Aspirin, a commonly used anti-inflammatory agent, was used to treat the induced manic-like symptoms and inflammation. Concentrations of a spectrum of inflammatory cytokines were examined by enzyme-linked immunosorbent assay in both plasma and brain tissues, and expression of Toll-like receptors 3 and 4 were determined in rat brains. Locomotor activity was monitored with open-field test to assess the effects of ouabain challenge and to evaluate the treatment efficacy of aspirin. Ouabain administration recapitulated many mania-like features such as increased stereotypic counts, traveling distance in open-field test, and decreased expression of brain-derived neurotrophic factor, interferon gamma, and Toll-like receptor 3, which were frequently found in patients with BD. These abnormalities could be partially reversed by aspirin. Our findings suggest that aspirin could be used as a promising adjunctive therapy for BD.

## Introduction

Bipolar disorder (BD) is a chronic, severe, and disabling medical condition, which frequently associates with high levels of morbidity and mortality (1). The lifetime prevalence is estimated as high as 2.4% (2). Numerous factors including genetics, oxidative stress, and environmental interaction predispose toward the pathogenesis of BD, but the precise pathological etiology remains enormously complex and debatable. Mood stabilizers and antipsychotics are first-line agents for BD with poor tolerance and high rates of treatment resistance. In addition, their effects are mostly palliative, which do not alter the overall prognosis.

Accumulating evidence has demonstrated that a high co-occurrence of inflammatory comorbidities with BD and immune dysfunction is emerging as a strong predisposition factor for this association (3–5). Many inflammatory comorbidities are associated with BD, such as systemic lupus erythematosis (6), autoimmune thyroiditis (7), psoriasis (8), Guillain-Barré syndrome, autoimmune hepatitis, multiple sclerosis (9), obesity, atherosclerosis and type II diabetes mellitus (10), rheumatoid arthritis (11), migraines, and inflammatory bowel disease (12, 13). In BP patients, levels of many circulating pro-inflammatory molecules were elevated, and inflammation was augmented in the brains (4). Accordingly, altered inflammatory cytokines in plasma have also been described during manic or depressive episodes (14). In addition, postmortem study has revealed increased transcripts of interleukin (IL)-1β, IL-1 receptor, myeloid differentiation factor 88, nuclear factor-kappa B subunits, and over-activated astrocytes and microglia in BP brains (15). In line with these observation, lithium and valproic acid have been demonstrated to exert mood stabilizing effects partially through immune system modulation (16, 17). As a result, BP itself is recently proposed as an inflammatory condition, and the recurrent mood episodes may represent fluctuating inflammatory states (13).

Toll-like receptors (TLRs) are important mediators of inflammatory responses and expressed on membranes or organelles of microglia. TLRs recognize endogenous molecules, referred to as danger-associated molecular patterns, which are typically sequestered from the immune system but released during tissue pathology (18). Among the TLR family, TLR3 and TLR4 in CNS are of particular interest, which can activate interferon (IFN) regulatory factor 3 and induce IFN-β production, followed by a phase of IFN-dependent gene expression, while the other family members of TLRs do not activate IFN regulatory factor 3 pathway (19). Furthermore, real-time PCR of TLRs 1–10-in cultured human astrocytes showed that TLR3 expression rapidly increased upon exposure to inflammatory cytokines IFN-γ, IL-1β, and IFN-β (20). Although increased peripheral TLR4 responses have been also reported in BD subjects (21), no further functional study was performed along this line.

Aspirin is a nonsteroidal anti-inflammatory drug, which suppresses the inflammatory response and reduces levels of inflammatory biomarkers such as C-reactive protein, tumor necrosis factor-α, and IL-6 (22). It can also reduce oxidative stress and protect against oxidative damage. There is some preclinical and clinical evidence suggesting beneficial effects for aspirin in mood disorders (23). Recently, anti-inflammatory regents including aspirin are proposed to have a moderate antidepressant effect in the treatment of BD (12), which is also confirmed in a phase IIA clinical trial (24). However, the underlying cellular mechanism remains unclear.

In the present study, ouabain was injected intra-cerebroventricularly to induce abnormally manic-like symptoms in rats (25, 26), which were followed with administration of aspirin. Animal behaviors were monitored before and after ouabain challenge and also after aspirin treatment. Concentration of inflammatory cytokines and expressing profiles of TLR3 or TLR4 were determined in plasma and brains of rats. Our data showed that ouabain injection reliably induced manic-like symptoms in rats with a spectrum of features similar with BD. Aspirin partially reversed abnormalities presented by the rats through upregulated expression of IFN-γ and TLR3 in brains. This study demonstrates that aspirin may have a beneficial impact in the treatment of BD.

## Methods

### Animals

Thirty adult male Sprague-Dawley rats (250–350 g) were purchased from Hunan Slack Scene Of Laboratory Animal Co., Ltd (Changsha, China). During the experiment, rats were housed individually in a room with a 12-h light/dark cycle (light on at 7:00 am) and environmental temperature of 25°C with 50–60% humidity with *ad libitum* access to food and water in the experimental animal center of the Second Xiangya Hospital. All the animals were habituated for at least 1 week before any manipulation. All the described procedures were approved by the Institution of Animal Care and Use Committee of The Second Xiangya Hospital (protocol number: 2015.014) and adhered to the Guide for the Care and Use of Laboratory Animals. Every effort was made to minimize animal suffering and the number of animals used.

### Surgical Procedure

Ouabain injection was performed as previously report (25, 27). Briefly, after intraperitoneal anesthetization with 10% chloral hydrate (3 ml/kg), animals were fixed in a stereotaxic apparatus (Narashiga, Japan). A longitudinal incision was made along the midline of scalp, and a 9-mm guiding cannula (27 gauge) was placed at the coordinates of 0.9 mm posterior to Bregma, 1.5 mm right from the midline, and 1.0 mm above the lateral brain ventricle (right side). Through a 2-mm hole made at the cranial bone, a cannula was implanted 2.6 mm ventral to the superior surface of the skull and fixed with jeweler acrylic cement.

### Treatment

All animals were randomly assigned into three groups. Rats in the first and second groups received a single intra-cerebroventricular (ICV) injection of 5 μl of ouabain (10^−3^ M) dissolved in artificial cerebrospinal fluid (aCSF), whereas the animals in the third group received an injection of 5 μl aCSF after surgery. From the second day following the injection of ouabain or aCSF, rats in the first group were treated for 1 week with aspirin (50 mg/kg), while those in the second and third groups were treated for 1 week with equal volume of saline by gavage. Three groups were categorized as follows: ouabain ICV + aspirin IG (OUA + APC), ouabain ICV + saline IG (OUA + SAL), and aCSF + saline IG (aCSF + SAL).

### Open-Field Test

Locomotor activities of the animals were assessed using the paradigm of open-field test as previously reported (25). The task was performed in a 43.2 × 43.2 × 30.5 – cm^3^ box on two occasions: immediately or 1 week after drug injection. The floor of the box was divided into nine equal rectangles with black lines. The animals were gently placed in the center area and free to explore the field for 5 min. All of the movements were tracked by an overhead camera. The total travel distance was analyzed by Computer-assisted Tracking Software (ENV-515-16, MED Associates, Inc). Rearing activity was monitored and videotaped automatically as main readout of stereotypic behavior. Rats were decapitated immediately after the last evaluation, and prefrontal cortices were dissected, snap frozen, and stored at -80°C until analysis.

### Measurement of Cytokines and Brain-Derived Neurotrophic Factor

Enzyme-linked immunosorbent assay (ELISA) was used to determine the concentrations of various cytokines and brain-derived neurotrophic factor (BDNF) as previously reported (28). Blood samples were obtained from the rats and centrifuged at 1,000×g at 4°C for 15 min. The supernatant were carefully taken and stored at −20°C. Prefrontal cortices from each animal were homogenized in phosphate buffer solution followed by centrifuge at 5,000 × g for 5 min at 4°C. The pellets were discarded, and the supernatant were stored at −20°C. The expressing levels of C-reactive protein (CRP), IL-1β, IL-2, IL-6, IL-10, INF-γ, tumor necrosis factor alpha (TNFα), prostaglandin E_2_ PGE-2, as well as BDNF and PGE-2 were subsequently measured by ELISA. Briefly, microtiter plates (96-well) were coated for 24 h with individual sample diluted with sample diluent, and a standard curve ranging from 0 to 320 pg/ml for each cytokine was plotted. The plates were then washed four times with sample diluent, followed by incubation with corresponding antibodies against each cytokine for 3 h at room temperature. After three times washing (with sample diluents), the plates were incubated with specified secondary antibody conjugated with peroxidase at room temperature for 30 min. Then peroxidase conjugated with streptavidin and reaction substrate was introduced for 5 min followed with the stop solution. Absorbance at 450 nm was read, and the amount of each cytokine was determined. Total protein was calculated by Lowry’s method using bovine serum albumin as a standard. All the ELISA kits were purchased from Wuhan USCN Business Co., Ltd.

### Expression of Toll-Like Receptors 3 and 4 in Frontal Cortex

Real-time reverse transcriptase PCR was used to determine the expression of TLR3 and TLR4 in frontal cortices of the rats. Standard protocols were used for total RNA extraction, complementary DNA (cDNA) synthesis, and PCR. cDNA sequence was obtained from Genebank at National Center for Biotechnology Information (www.ncbi.nlm.nih.gov). Primer sequences were designed using Operon Oligo Analysis Tool (https://www.eurofinsgenomics.com/en/resources/tools/pcr-primer-design/), and the sequence specificity was validated with the basic local alignment search tool. Primers were purchased from Invitrogen, and the specificity was verified by melt curve analyses. The following primers were used: TLR3, F: 5’-CTGGGTCTGGGAGCATTTC-3’, R: 5’-GCGGGTCTTTCAGTAGGTG-3’; TLR4, F: 5’-ATGAGGACTGGGTGAGAAAC-3’, R: 5’-ACCAACGGCTCTGGATAAAG-3’. PCR amplification of cDNA was performed using the SYBR Green PCR Kit (Thermo, USA). The quantity of PCR product was monitored in real time using the MyiQ Single-Color Real-Time PCR Detection System (Bio-Rad, Hercules, CA, USA). Relative gene expressing abundance was calculated as the ratio of TLR3 or TLR4 against actin.

### Western Blot Analysis

For immunoblotting, frontal cortices were homogenized with lysis buffer (Cell Signaling Technology) containing complete protease and phosphatase inhibitors (Roche) and centrifuged at 12,000×g for 15 min at 4°C. The protein concentration in supernatant was determined using bicinchoninic acid protein assay kit (Thermo Scientific, USA). Equal amounts of protein were fractionated by sodium dodecyl sulfate polyacrylamide gel electrophoresis and transferred to nitrocellulose membranes. The membranes were blocked and incubated with primary antibodies overnight at 4°C, incubated with horseradish peroxidase-conjugated secondary antibody (Invitrogen), and developed using an enhanced chemiluminescence kit (Millipore). The following primary antibodies were used: TLR3 (Bioworld 1:1,000, BS6749), TLR4 (Abcam, 1:500, Ab22048), and glyceraldehyde 3-phosphate dehydrogenase (Cell Signalling Pathway, 1:1,000, CST #5174). After three washing times with 0.01-M Tris-buffered saline, the membranes were probed with horseradish peroxidase-conjugated secondary antibodies at room temperature for 1 h and then developed with enhanced chemiluminescence kit (ECL-plus, Thermo Scientific, USA).

### Statistical Analysis

Behavioral and biochemical data were presented as mean and standard error of the mean. The equality of variance in multiple data groups was assessed with Levene’s test. Statistical difference was determined with one-way ANOVA, which was followed by least significant difference (LSD)-t test or Tamhane test. *P* < 0.05 was considered as statistical significance threshold.

## Results

### Aspirin Facilitated the Amelioration of Hyperactivity Caused by Ouabain Intra-Cerebroventricular Injection

**Figure 1:**
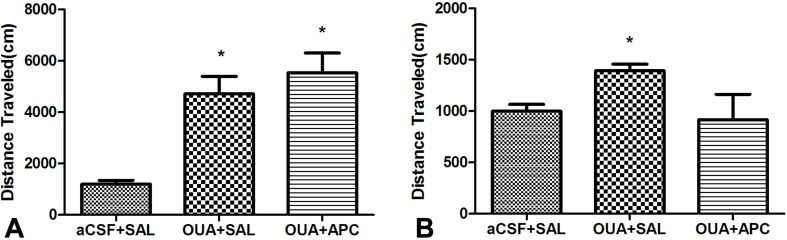
Traveling distance was analyzed prior to and after aspirin treatment. **(A)** Data analysis for the first open-field test, *p < 0.05 vs. aCSF + SAL group (ANOVA followed by Tamhane test, F = 16.653, *p* < 0.001). **(B)** Data analysis for the second open-field test, *p < 0.05 vs. aCSF + SAL group (ANOVA followed by Tamhane test, F = 2.926, *p* = 0.075).

**Figure 2:**
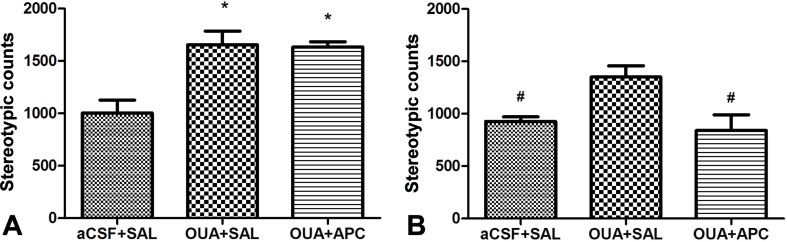
Stereotypic counts of rats in three groups before and after aspirin treatment. **(A)** Data analysis for the first open-field test, *p < 0.05 vs. aCSF + SAL group (ANOVA followed by LSD-t test, F = 11.779, *p* < 0.001). **(B)** Data analysis for the second open-field test, ^#^ *P* < 0.05 vs. OUA + SAL group (ANOVA followed by Tamhane test, F = 6.518, *p* < 0.01).

Previous studies have demonstrated that ICV injection of OUA in rats could lead to hyperactive locomotion, a phenotype compatible with the manic episode of BD patients. Open-field test is most widely used to assess this phenotype due to its supreme sensitivity and consistence compared with automated activity monitor (27). We hence performed open-field test to examine effects of aspirin to the behavioral phenotypes caused by OUA. Consistently, OUA immediately increased the traveling distance and stereotypic counts in OUA + SAL and OUA + APC groups after the ICV administration (**Figure 1**). Aspirin treatment modestly reduced stereotypic counts compared with the OUA + SAL group (**Figure 2B**), which indicates an attenuation of increased stereotypic counts. In line with the reports by El-Mallakh et al., rats in the OUA + SAL group still presented hyperactivity during the second open-field test indicating that the effects of OUA are potent and can last at least for 1 week (**Figure 2A**). There was no effect on distance traveled between OUA + SAL and OUA + APC groups in the second open-field test, indicating that aspirin may only have modest effects, although the role of novel environment cannot completely be ruled out (**Figure 2A**).

### Expressing Profiles of Brain-Derived Neurotrophic Factor and Pro-Inflammatory Cytokines

**Figure 3:**
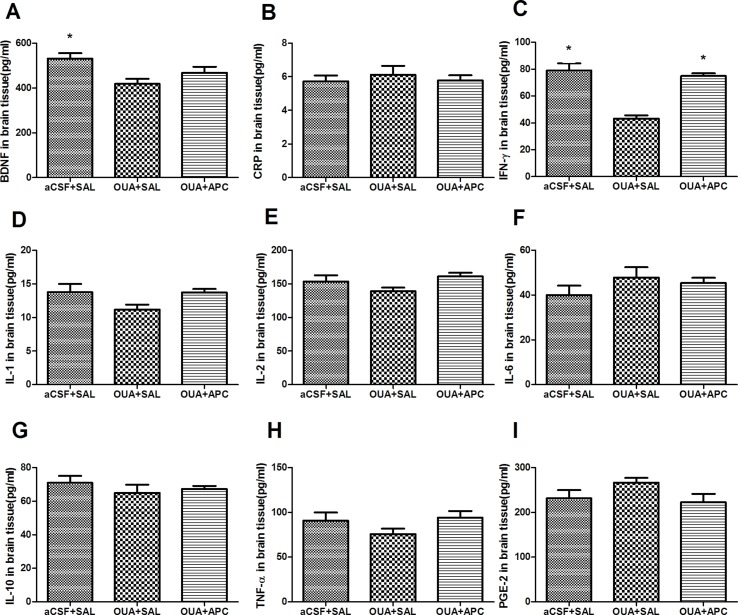
Expressing levels of BDNF and various cytokines in brain tissue after ICV administration of OUA and aspirin treatment. **(A–C)**, BDNF, CRP and IFN-γ; **(D–G)**, IL-1β, 2, 6, and 10; **(H-I)**, TNF-α and PGE-2. *P < 0.05 vs. OUA + SAL group, according to ANOVA followed by the LSD-*t* or Tamhane test (BDNF: F=5.110, *p* < 0.05; INF-γ: F=29.687, *p* < 0.001).

We next asked whether OUA injection could alter expression of BDNF and activate cascades of cytokines in both brain and periphery. In line with previous report (28), the expressing levels of cytokines CRP, IL-1β, IL-2, IL-6, IL-10, INF-γ, TNFα, as well as BDNF and PGE-2 in plasma did not show any difference among groups (data not shown). Notably, BDNF and INF-γ levels in prefrontal cortices of the aCSF + SAL group were higher than those in the OUA + SAL group, indicating that ICV administration of OUA decreased BDNF and INF-γ levels in brain tissue (**Figures 3A, C**). Intriguingly, INF-γ levels in brain tissue of aspirin treated rats increased compared with those in the OUA + SAL group, which indicated that aspirin reversed the reduction of INF-γ (**Figure 3C**). Apart from this observation, no difference was detected for the expressing profiles of cytokines such as CRP, IL-1β, IL-2, IL-6, IL-10, TNFα, and PGE-2 in brain tissues among groups (**Figures 3B, D–I**).

### Expression of Toll-Like Receptors 3 and 4 in Prefrontal Cortex

**Figure 4:**
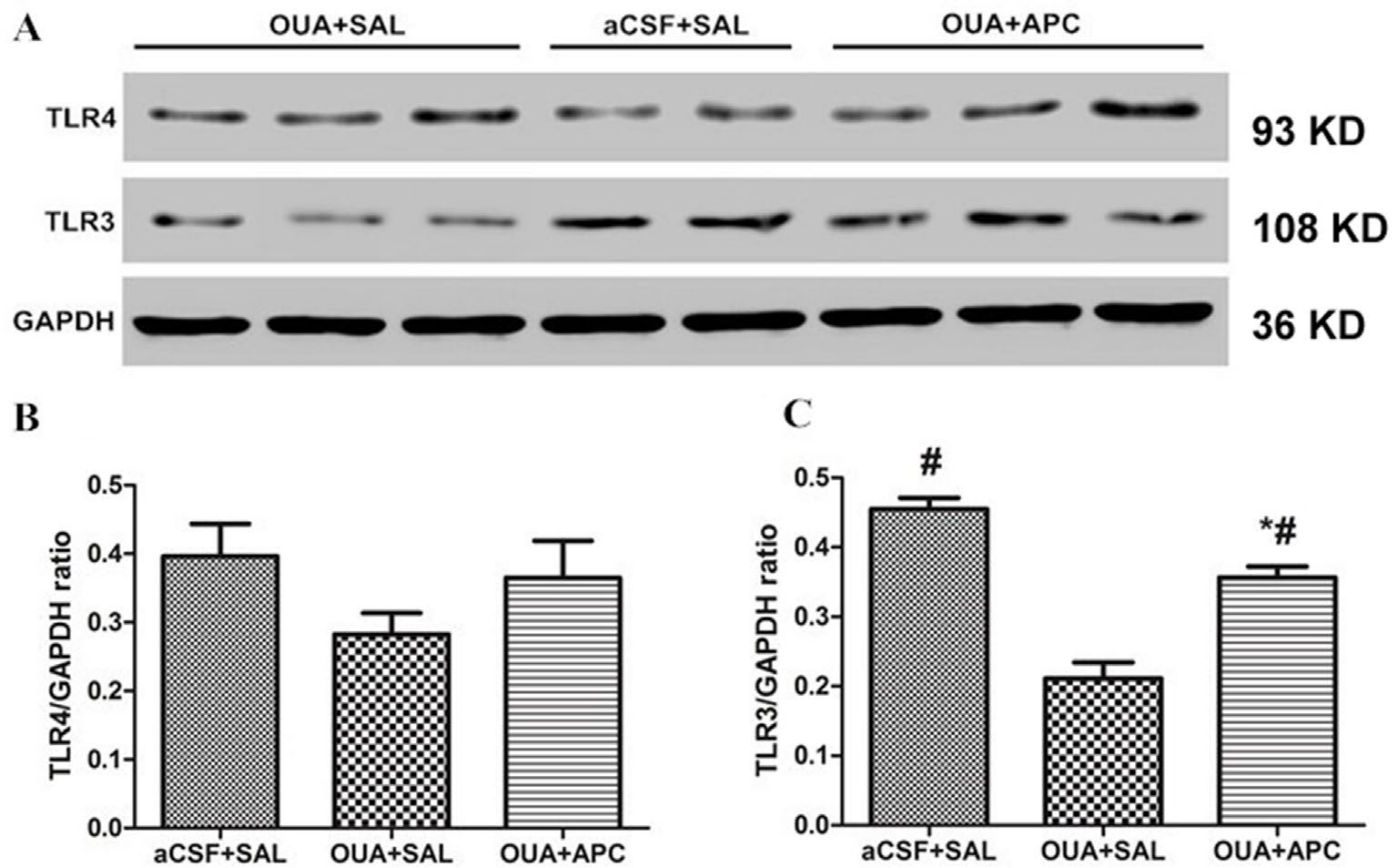
TLR4 and TLR3 protein levels in brain tissue after ICV administration of OUA and aspirin treatment. **(A)** Western blotting results; **(B–C)** Statistical analysis. *P < 0.05 vs. aCSF + SAL group, ^#^ *p* < 0.05 vs. OUA + SAL group, according to ANOVA followed by the LSD-*t* test (TLR3 protein levels: F = 42.64, *p* = 0.000).

TLRs are important modulators of immune homeostasis that facilitate the production of proinflammatory cytokines/chemokines including IFN-γ. Recently, a growing body of evidence indicates that TLR3 and TLR4 may be involved in psychosis with immune dysfunction including major depressive disorders (29) and BD (30). We therefore performed real-time reverse transcriptase PCR and Western blotting experiments to determine the expression of TLR3 and TLR4 in prefrontal cortices. We did not find any difference for the messenger RNA (mRNA) expression levels of TLR3 and TLR4 among the three groups; however, TLR3 protein levels in frontal cortex were overtly decreased in the OUA + SAL group, and aspirin treatment significantly increased TLR3 protein levels compared with that of the OUA + SAL group (**Figures 4A, C**). These results indicate that aspirin could reverse the reduction of TLR3 protein levels caused by OUA injection. Interestingly, TLR4 protein levels did not show any difference between the three groups (**Figures 4A, B**).

## Discussion

BD is a refractory and relapsing illness with strong components of innate immune dysfunction. However, previous studies mainly focus on depression phase of BD, and little is known about the dysfunctional immunomodulation in manic phase of the disease. In the present study, we recapitulated manic-like behaviors in rats by ICV administration of ouabain, a potent Na^+^/K^+^-ATPase inhibitor. Ouabain injection successfully induced mania-like behaviors such as increased stereotypic counts and traveling distance that associated with aberrant expression of BDNF, INF-γ, and TLR3 in both plasma and prefrontal cortex. Importantly, aspirin treatment not only reversed the increased stereotypic counts of the rats but also increased expressing levels of BDNF, INF-γ, and TLR3 in rat plasma and brains. Our data suggest that inflammation may be closely involved in a subpopulation of BD patients and aspirin treatment could provide beneficial effects to BD patients with prominent inflammatory comorbidities.

In our study, ICV administration of OUA, a potent Na^+^/K^+^-ATPase inhibitor, caused mania-like behaviors such as increased stereotypic counts and traveling distance. Previous studies showed that ouabain-induced hyperlocomotion occurred immediately and could persist for 7 days after a single ICV administration (25, 26, 28, 31). In addition to the behavioral phenotypes, rats with manic-like symptoms also displayed decreased protein levels of BDNF, INF-γ, and TLR3 in brain tissue. Largely due to the potent modulation of inflammation and neurogenesis, BDNF is closely involved in neuroplasticity and has long been proposed as a potential biomarker for many mental disorders. In line with our findings, expression of BDNF in hippocampus and amygdala also decreased in rats after ouabain administration that could be prevented and reversed by lithium (31). Importantly, manic patients also exhibited decreased BDNF level in serum that was associated with acute mood episodes in both medicated (32) and unmedicated patients (33). Interestingly, accumulating evidence has demonstrated that mood-stabilizing drugs, including lithium and valproate, have important neuroprotective roles in acute and chronic treatment (34), and one of the underpinning mechanisms is to increase BDNF levels in both serum and brain (35). As a response to treatment, manic patients often experience a sharp increase of BDNF in serum prior to the resolution of acute episode (36). This seems to support the concept that the main determinant related to lower BDNF levels in depression and mania is the presence of symptoms, not medication status. However, it must be noted that BDNF precisely modulates synaptic connection and signal transmission that helps to maintain structural and functional homeostasis of brain. It is sensitive in many neuropsychiatric conditions and thus lacks specificity to BD.

IFN-γ is a pleiotropic cytokine that induces antiviral, antiproliferative, and immune-modulatory effects in numerous inflammatory diseases (37). IFN-γ has been proposed to involve in the pathophysiology of BD, but the underpinning mechanism remains unclear. Previous clinical research revealed inconsistent expression of circulating IFN-γ in serum. Hope et al. did not find any alteration of IFN-γ in serum of BD patients (38), while upregulated expression of IFN-γ was also reported (39, 40). In the present study, we have detected significant decrease of IFN-γ in serum of BD patients. In agreement with our findings, stimulation of lymphocytes *in vitro* also led to a lower release of IFN-γ (41). Because IFN-γ can enhance neurogenesis in dentate gyrus of adult mice and promote spatial learning and memory performance, we propose a lower concentration of IFN-γ may have deleterious effects to the integrity of brain function of mice (42), although the precise underpinning mechanism needs to be elucidated further.

TLR3 is a key member of TLR family and plays an important role in the developmental patterning of innate immunity and autonomously regulates the establishment of neural network (43). Upon exposure to its specific ligand polyinosine:polycytidylic acid, TLR3 can rapidly cause growth cone collapse and inhibit neurite extension independent of nuclear factor kappa-light-chain-enhancer of activated B cells. In CNS, TLR3-mediated activation of astrocytes led to a marked induction of the enzyme indoleamine 2,3-dioxygenase, which acted as a local immune-suppressive factor (44). TLR3 also had a protective role in experimental autoimmune encephalitis due to increased expression of IFN-β (45). In cultured human astrocytes, TLR3 expression rapidly increased upon exposure to IFN-γ, IL-1β, and IFN-β (20). In our study, we did not detect any changes in TLR3 mRNA but a reduced expression of TLR3 protein. It should be noted that mRNA abundance is not always paralleled with the expression of corresponding proteins and discrepancy frequently occurs. For example, Abdi et al. has detected very low level of TLR3 mRNA but strong protein translation in human multiple myeloma cells. On the contrary, abundant mRNA transcripts of TLR5 were confirmed, but there is almost no protein expression (46). Similarly, Arvaniti et al. found that some B-cell chronic lymphocytic leukemia cells do not express TLR6 protein in spite of a high mRNA level and a high expression of proteins for TLR2 and TLR8 in spite of a low mRNAs (47). We assume that the reduced TLR3 protein could be subsequent outcome of reduced IFN-γ, although the precise mechanism needs to be further illustrated.

In the present experimental paradigm, we did not find any change of CRP, IL-1β, IL-2, IL-6, IL-10, INF-γ, TNFα, as well as BDNF and PGE-2 in rat plasma. Consistently, no alteration was detected for the concentration of CRP, IL-1β, IL-2, IL-6, IL-10, TNFα, and PGE-2 in rat brains challenged by our or aspirin. Similarly, Tonin et al. assessed concentrations of various cytokines (IL-1β, IL-6, IL-10, TNF-α, and cytokine-induced neutrophil chemoattractant 1) in distinct brain structures (hippocampus, striatum, frontal cortex, and amygdala), serum, and cerebrospinal fluid (CSF) of rats subject to ouabain administration and only found decreased IL-6 in striatum (28). These findings highlight that despite the observed behavioral phenotypes, ouabain administration cannot produce overt alteration of most pro-inflammatory factors commonly occurring in BD patients. This may largely limit its usage as a generalized approach to mimic human BD. On the other hand, no detectable changes of cytokines in periphery also indicate that altered IFN-γ and TLR3 in rat brains are highly likely the outcomes of ouabain injection, although the influence of surgical procedure cannot be completely ruled out.

Anti-inflammatory treatment is rapidly arising as a new augmentation therapy for BD patients due to the high proportion of medical comorbid conditions. Aspirin is a particularly promising candidate, which has been well established in various clinical settings and is well tolerated even in long-term use. Importantly, it is well absorbed and brain penetrant. Low dosage of aspirin preferentially inhibits cyclooxygenase 1, which further blocks inflammatory cascades by conversion of arachidonic acid to prostaglandins and thromboxane A2. In clinical practice, compelling evidence has confirmed that aspirin can inhibit the production of pre-inflammatory cytokines, such as CRP and tumor necrosis factor-α (22). In addition, it can also downregulate oxidative stress and protect against oxidative damage (Mendlewicz et al., 2006) and acute cerebral infarction (48). Importantly, in patients with depression, aspirin successfully reduced oxidative stress (49) and promoted actions of antidepressants (50). In our study, we have demonstrated that aspirin successfully reduced manic-like behaviors in rats and elevated the expression of INF-γ and TLR3 in brain tissue. As a matter of fact, low-dose aspirin could produce a statistically significant duration-independent reduction in the relative risk of clinical deterioration in BD subjects treated with lithium (23). Epidemiological data also confirm that aspirin protects against depression in older men with elevated levels of homocysteine (51). Recently, a preliminary study of a phase IIA clinical trial has convincingly demonstrated a main effect of low-dose aspirin for BD patients treated with or without minocycline (24). Because low dosage of aspirin is mostly benign and affordable, an adequately-powered study in large-scale will help consolidate the concept of aspirin as an adjunctive treatment option for BD or even other relevant diseases such as schizophrenia and Alzheimer’s disease. However, a major limitation of this experiment is that animals only received a single dose of aspirin that lasts for 1 week. Treatment with multiple dosages for longer time may help to precisely reveal roles of aspirin and inflammatory modulation in ouabain-induced manic-like symptoms in rats.

In summary, we have demonstrated that rats with ouabain ICV injection display reduced expression of BDNF, INF-γ levels, and TLR3 in brain tissues, and aspirin supplement can elevate the expression of INF-γ and TLR3. We propose that aspirin may be of potential benefit for adjunctive treatment of BD.

## Data Availability

All datasets generated for this study are included in the manuscript and the supplementary files.

## Ethics Statement

All the described procedures were approved by the Institution of Animal Care and Use Committee of The Second Xiangya Hospital (Protocol number: 2015.014) and adhered to the Guide for the Care and Use of Laboratory Animals.

## Author Contributions

All authors listed have made substantial, direct, and intellectual contribution to the work and approved it for publication.

## Funding

This research was supported by grants from National Key R&D Program of China (2016YFC1306900), National Natural Science Foundation of China (81622018), and Key Research and Development Program from Hunan Province (2018DK2011).

## Conflict of Interest Statement

The authors declare that the research was conducted in the absence of any commercial or financial relationships that could be construed as a potential conflict of interest.
